# Influence of calcination temperature on the properties and photocatalytic efficiencies of BiFe_0.9_Cu_0.1_O_3_ nanoparticles

**DOI:** 10.1038/s41598-025-34901-8

**Published:** 2026-01-30

**Authors:** Shimaa R. Abdel-Kader, Ahmed M. El-Awad, M. A. Abdel Rahim

**Affiliations:** 1 Chemistry Department, Faculty of Science, Assiut University, Assiut, 71516 Egypt; 2 Physics Department, Faculty of Science, Assiut University, Assiut, 71516 Egypt

**Keywords:** BiFe_0.9_Cu_0.1_O_3_ nanoparticles, Structural, Optical, Photocatalytic activity, Chemistry, Materials science, Nanoscience and technology

## Abstract

BiFe₀.₉Cu₀.₁O₃ nanoparticles were synthesized via a simple solution combustion method, and the effect of calcination temperature (500–800 °C) on their properties and photocatalytic performance was systematically investigated. The nanoparticles were characterized using XRD, FTIR, FE-SEM, BET surface area analysis, XPS, UV-Vis diffuse reflectance, photoluminescence, and VSM. XRD analysis confirmed a primary rhombohedral BiFeO₃ phase alongside a secondary orthorhombic Bi₂Fe₄O₉ phase. The results revealed a complex interplay between calcination temperature and material properties: the crystallite size increased with temperature up to 700 °C, then decreased slightly. At the same time, the specific surface area was maximized at 500 °C. The optical band gap reached a minimum of 3.30 eV at 500 °C, then widened at higher temperatures. VSM measurements revealed ferromagnetic behavior at room temperature, with the saturation magnetization peaking at 600 °C due to the suppression of the spin spiral structure and the presence of oxygen vacancies. The photocatalytic activity for methylene blue (MB) degradation under visible light was maximized at 500 °C, achieving 72.75% degradation efficiency after 160 min. This optimal performance is attributed to the synergistic combination of the highest specific surface area, a favorable band gap, and a high concentration of defect sites that suppress charge carrier recombination. The optimal sample demonstrated good stability and reusability over five cycles. Scavenger tests indicated that the hydroxyl radicals (HO^•^) were the primary reactive species. The results confirm that solution combustion is an effective route for producing Cu-doped BFO nanoparticles, with the calcination temperature being a critical parameter for optimizing their photocatalytic performance.

## Introduction

Dyes are a significant source of water pollution, and their treatment is an area of intense research. However, various biological and chemical treatment methods have been suggested. One of the most effective ways to remove these pollutants may be to employ semiconductor compounds with photocatalytic activity. Among various materials, binary metal oxides, such as TiO_2_, have been extensively studied due to their excellent photocatalytic properties^1^. However, the relatively wide band gap of TiO_2_ (3–3.5 eV) limits its applicability under visible light^2^, which is harmful to human health due to its large band gap. As an alternative to binary metal oxides, ternary oxide nanoparticles show better stability in aqueous conditions^3^. More importantly, however, ternary metal oxides have some significant advantages over binary metal oxides in various applications. Bismuth ferrite (BiFeO₃) is a multiferroic perovskite material with a narrow band gap (~ 2.1 eV), demonstrating significant potential as a photocatalyst for environmental remediation and sustainable energy applications. Its photocatalytic capabilities include dye degradation, air purification, wastewater treatment, and hydrogen generation, all driven by its ability to harness visible light^4^. Additionally, Multiferroic materials have garnered significant interest due to their potential in emerging multifunctional technologies, including spintronics, optoelectronics, transducers, multi-state memory, data storage media, sensors, and high-frequency devices^5–9^. Among them, the multiferroic material bismuth ferrite, with the chemical formula BiFeO_3_ (BFO), has received considerable attention due to its multiferroic properties at room temperature. The crystal structure of BFO is in the form of a distorted rhombohedral perovskite structure with the point group R3C at room temperature^9,10^. Furthermore, BFO nanoparticles have garnered considerable attention due to their unique electrical, optical, and magnetic properties, and they have potential applications in memory devices, storage, communication, and smart devices^11–14^. As a photocatalyst, BFO as a photocatalyst has been recognized due to its small band gap, non-toxicity, chemical stability, low cost, and good response to visible light irradiation^11–15^.

In recent years, various researchers have synthesized BFO with different methods. Biasotte et al.^16^ prepared single-phase BFO using the microwave-hydrothermal method and compared it with the conventional solid-state reaction method. Hamed Maleki et al.^17^ prepared bismuth ferrite nanocrystallites by the co-precipitation method. The phase analysis, structural, and magnetic properties of the product were studied. Furthermore, Mostafavi et al.^18^ synthesized the multiferroic bismuth via the conventional solid-state reaction method. They observed numerous secondary phases in conjunction with the primary phase of BFO in their analysis. Generally, the photocatalytic activity of photocatalysts is mainly influenced by the rate of electron-hole recombination, specific surface area, crystallite/grain size, defect sites, morphology, and crystal structure. These factors can be controlled by doping, calcination temperature, and preparation technique.

However, the quick recombination of electrons and holes is a major problem, causing a decrease in photocatalytic activity. To solve this problem, numerous researchers have focused on enhancing this photocatalytic activity with doping by various transition and rare-earth metals such as Cu, Al, Ni, Mg, Cd, Ce, Sr, etc^15,19–22^. Doping with transition metal ions on the semiconductor photocatalyst surface has been used to increase the lifetime of charge carriers and act as electron traps to prevent the recombination of photoinduced charge carriers. Therefore, doping Cu^2+^ ions in BFO is an interesting research area for its photocatalytic applications. The Cu^2+^ has a smaller ionic radius than Fe^3+^, and when it substitutes for Fe^3+^ introduces local lattice distortions. These distortions can lead to a smaller particle size and higher surface area, both beneficial for photocatalysis. Also, the Cu^2+^ ions can act as electron or hole traps. This reduces the electron-hole recombination rate and increases the lifetime of charge carriers, which enhances photocatalytic efficiency. Furthermore, the choice of Cu (II) ion doping was made to obtain higher concentrations of oxygen vacancies, which significantly influence the photocatalytic activity of catalysts through retarding the recombination of e^−^ - h^+^ pairs.

However, few reports exist on Cu-doped BFO and its photocatalytic applications. Samran et al.^23^ studied the effect of Cu-doped BFO on photocatalytic activity. The authors observed that the Cu-doped BFO with 0.1 mol% has the highest photocatalytic activity for the degradation of MB aqueous solution under visible light irradiation. On the other hand, the calcination temperature is another important parameter for improving photocatalytic properties. Ritesh et al. studied the effect of calcination temperature on the structural, morphological, and optical properties of BFO nanoparticles^24^. Moreover, different techniques are employed for the synthesis of BFO nanomaterials, including the hydrothermal method, electrospinning, solid-state method, sol-gel method, co-precipitation technique, and combustion technique. Among these techniques, the combustion method is speedy, uncomplicated, and gives a homogeneous product. However, not many reports are available on the use for the synthesis of BFO nanoparticles by the combustion technique.

In the present work, BFO doped with 0.1 mol% Cu has been successfully synthesized using the solution combustion (SC) technique, and the final product was calcined at different temperatures (500, 600, 700, and 800 °C). The BiFe_0.9_Cu_0.1_O_3_ was extensively characterized by using XRD, FTIR, FE-SEM, VSM, XPS, Diffuse reflectance, photoluminescence, BET surface area, and UV-Visible analysis. Furthermore, the photocatalytic activities of the samples studied were examined by the photodegradation of the MB dye solution under visible light irradiation. It is observed that BiFe_0.9_Cu_0.1_O_3_ calcined at 500 ͦ C has a smaller crystallite size, lower optical band gap, and higher specific surface area, which exhibits higher photocatalytic activity, nearly 72.75% at 160 min.

## Experimental procedure

### Materials and preparation

BiFe₀.₉Cu₀.₁O₃ nanoparticles were synthesized via a solution combustion method using high-purity (99.9%) Sigma-Aldrich reagents. Stoichiometric amounts of bismuth (III) nitrate pentahydrate (120 mmol), iron (III) nitrate nonahydrate (108 mmol), copper (II) nitrate trihydrate (12 mmol), urea (300 mmol), and glycine (201.68 mmol) were each dissolved separately in distilled water in ratios 1:0.9:0.1:2.5:1.7, respectively. Due to the poor solubility of bismuth nitrate, a few drops of diluted nitric acid (HNO₃) were added to its solution to achieve clear dissolution. The individual metal nitrate solutions were then combined, and a mixed fuel solution of glycine and urea was introduced dropwise into the mixture. The resulting aqueous solution was stirred at 120 °C for approximately 20 h to ensure homogeneity, maintaining a constant volume of 500 mL. Following the complete evaporation of water, a self-propagating combustion reaction occurred, transforming the mixture into a voluminous, foam-like residue that yielded a fine precursor powder. Finally, the as-synthesized powder was calcined at different temperatures (500, 600, 700, and 800 °C) for 3 h to obtain the final crystalline products.

### Characterization and equipment

The structural properties of the as-synthesized powder and samples calcined at 500, 600, 700, and 800 °C for 3 h were analyzed using a Phillips PW 1700 X-ray diffractometer (XRD) operating at 40 kV and 30 mA with Cu Kα radiation (λ = 1.5406 Å). XRD patterns were collected over a 2θ range of 10° to 80° at a scanning speed of 36°/min. Surface morphology was examined by field emission scanning electron microscopy (FESEM) using a ZEISS Gemini 450 instrument at an accelerating voltage of 10 keV, coupled with energy-dispersive X-ray spectroscopy (EDX) for elemental analysis. Fourier-transform infrared (FTIR) spectra were acquired in the range of 400–4000 cm⁻¹ using a ThermoScientific™ Nicolet™ iS™10 spectrometer.

The chemical composition and oxidation states of the BiFe₀.₉Cu₀.₁O₃ sample calcined at 600 °C were determined by X-ray photoelectron spectroscopy (XPS) on a Thermo Scientific K-ALPHA system. Measurements were performed using a monochromatic Al Kα source (0–1350 eV), a 400 μm spot size, a base pressure of 10⁻⁷ mbar, a pass energy of 200 eV, and a bandwidth of 50 eV. Magnetic properties were evaluated using a Lakeshore 7410 vibrating sample magnetometer (VSM) with applied fields ranging from 0 to 20 kG.

Specific surface area was determined by the Brunauer–Emmett–Teller (BET) method from the linear part of the nitrogen adsorption isotherm. Nitrogen adsorption-desorption isotherms were measured at 77 K using a Nova 3000 instrument (Quantachrome Instruments), with samples degassed at 250 °C for 2 h before analysis. Optical properties were investigated using a Thermo Scientific Evolution 220 UV-Visible spectrophotometer equipped with an ISA-220 integrating sphere, collecting diffuse reflectance spectra from 200 to 900 nm. The band gap energy was estimated from a Tauc plot derived from the reflectance data. Photoluminescence was investigated using a spectrofluorometer (FP-6300), 220 V and 50/60 HZ, 285 VA. Finally, the photocatalytic activity of the nanoparticles was evaluated by monitoring the degradation of methylene blue (MB) in aqueous solution under visible light irradiation.

### Photocatalytic activity experimentally

The photocatalytic performance of the as-synthesized and calcined BiFeO₃ nanoparticles doped with 0.1% Cu was evaluated by monitoring the degradation of methylene blue (MB) dye under visible light. The experiments were conducted under natural sunlight on clear, consecutive days in July and August 2024, between 11:00 AM and 3:00 PM. In a standard procedure, 50 mg of the catalyst was dispersed in 100 mL of an aqueous MB solution (15 mg/L). The suspension was first stirred in the dark for 60 min to establish adsorption-desorption equilibrium. To investigate the primary reactive species involved in the degradation mechanism, scavenger tests were performed by introducing 1 mmol of specific scavengers, isopropanol (IPA) for hydroxyl radicals (^•^OH), ethylenediaminetetraacetic acid disodium salt (EDTA-2Na) for holes (h⁺), and silver nitrate (AgNO_3_) for superoxide radicals (^•^O₂⁻), to the reaction mixture before illumination. Following this, the solution was exposed to sunlight, and approximately 5 mL aliquots were withdrawn at specific time intervals. The concentration of MB was determined by measuring the absorbance of the centrifuged samples using a Labomed UVD-2950 UV-Vis spectrophotometer.

## Results and discussion

### X-ray diffraction analysis

**Fig. 1:**
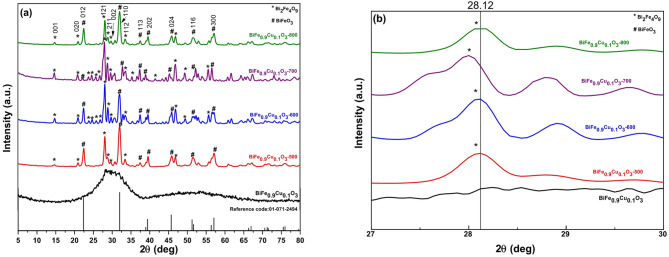
XRD patterns of (**a**) BiFe_0.9_Cu_0.1_O_3_ nanoparticles with different calcination temperatures; (**b**) the enlarged XRD patterns at 2θ = 27° to 30°.

The crystalline phases of the as-synthesized BiFe₀.₉Cu₀.₁O₃ nanoparticles and those calcined at 500, 600, 700, and 800 °C for 3 h were analyzed by X-ray diffraction, as shown in Fig. 1a, b. The XRD pattern of the as-synthesized powder indicates an amorphous nature, which crystallizes upon calcination into a multi-phase system. The diffraction patterns for the calcined samples show distinct peaks indexed to the (012), (110), (113), (202), (024), (116), and (300) crystal planes, confirming the formation of a primary crystalline phase of rhombohedral BiFeO₃ (JCPDS card no. 01-071-2494, space group R3c)^25^. Concurrently, only minimal traces of additional peaks around the (001), (020), (121), (211), (022), and (112) planes are attributed to the formation of a very small concentration of mullite phase Bi_2_Fe_4_O_9_ as a secondary orthorhombic phase (JCPDS card no. 00-025-0090)^26^. The absence of separate copper oxide peaks suggests that the Cu²⁺ ions have been incorporated into the BiFeO₃ lattice and/or the Bi₂Fe₄O₉ phase without forming a separate phase, likely due to their low concentration. Furthermore, the intensity of the major diffraction peaks increases and the full width at half maximum (FWHM) decreases with rising calcination temperature, indicating an improvement in crystallinity for both phases, which is consistent with other reports^25–27^.

A magnified view of the XRD patterns in the 2θ range of 27° to 30° is shown in Fig. 1b to examine the crystal structure in detail. This reveals that the position of the orthorhombic Bi₂Fe₄O₉ (121) peak shifts slightly toward lower 2θ values with increasing calcination temperature. This observed shift can be attributed to changes in lattice parameters at the phase interface and a reduction in lattice micro-strain and dislocation density.

Structural parameters, including the average crystallite size (D), micro-strain (ε), and dislocation density (δ), were calculated from the XRD data. For the rhombohedral structure (R3c), the average crystallite size (D, in nm) was determined using Scherrer’s formula^28^:

1 $$\:D=\frac{0.9\lambda\:}{\beta\:{cos}\theta\:}$$

where λ is the X-ray wavelength (1.5406 Å), θ is the Bragg diffraction angle (in degrees), and β is the full width at half maximum (FWHM) of the diffraction peak (in radians). The dislocation density (δ) and micro-strain (ε) for the as-synthesized and calcined BiFe₀.₉Cu₀.₁O₃ samples were calculated using the following equations^29^:

2 $$\delta = \frac{1}{{D^{2} }}$$

3 $$\:{\upepsilon\:}=\frac{\beta\:{cos}\theta\:}{4}$$

The calculated values of D, δ, and ε are listed in Table 1 as a function of calcination temperature. The results indicate that the average crystallite size (*D*) increased from 18.84 nm to 27.53 nm as the calcination temperature rose to 700 °C, followed by a slight decrease at 800 °C. In contrast, both the dislocation density (δ) and micro-strain (ε) exhibited a decreasing trend with increasing temperature, which can be attributed to particle agglomeration and crystallite growth.

Furthermore, the lattice parameters (a and c) for the rhombohedral crystal system (where a = b ≠ c) were estimated using the following relations:

4 $$\:\mathrm{a}=\frac{\lambda\:}{\sqrt{3}{sin}\theta\:}$$

5 $$\:\mathrm{c}=\frac{\lambda\:}{{sin}\left(\theta\:\right)}$$

The volume (V) of the unit cell was then calculated using the equation:

6 $$V{\text{ }} = {\text{ }}0.866{\text{ }}a^{2} c~$$

The specific surface area (SSA) was estimated by assuming the BiFe₀.₉Cu₀.₁O₃ nanoparticles to be spherical and uniform in size, using the following equation^30^:

7 $$\:SSA=\frac{6000}{\rho\:D}$$

where D is the average crystallite size in nm obtained from XRD, and ρ is the theoretical density of BiFe₀.₉Cu₀.₁O₃ (approximately 8.35 g/cm³).

**Table 1:** Crystallographic parameters of BiFe₀.₉Cu₀.₁O₃ samples calcined at different temperatures.

Sample	D (nm)	ε	δ ×10^− 3^ (nm^− 2^)	a (Å)	c (Å)	V (Å^3^)	c/a ratio	SSA (m^2^/g)
BiFeCuO-500	18.84	0.612	3.245	5.5812	13.879	374.43	2.486902	38.1
BiFeCuO-600	23.87	0.524	2.322	5.5917	13.840	374.78	2.475169	30.1
BiFeCuO-700	27.53	0.413	1.355	5.5821	13.996	377.69	2.507354	26.1
BiFeCuO-800	20.61	0.571	2.825	5.5704	13.863	372.54	2.488798	34.9

The data in Table 1 reveal several key trends in the structural evolution of the BiFeO₃ rhombohedral phase as the main phase with calcination temperature, which are crucial for interpreting their photocatalytic performance. Regarding crystallite size, strain, and defects, the average crystallite size (*D*) of the BiFeO₃ phase increases from 18.84 nm to 27.53 nm as the calcination temperature rises from 500 °C to 700 °C. This is a classic result of enhanced atomic diffusion and crystal growth at higher temperatures. Concurrently, the micro-strain (ε) and dislocation density (δ) decrease significantly over the same range. This inverse relationship is expected, as the relaxation of the crystal lattice during growth reduces internal strain and defect density. However, the subsequent decrease in crystallite size to 20.61 nm at 800 °C is a critical observation. This is unlikely to be true crystal dissolution and is more plausibly attributed to particle agglomeration or the enhanced growth of the secondary Bi₂Fe₄O₉ phase at the expense of the primary BiFeO₃ phase. Agglomerates can lead to broader XRD peaks, which Scherrer’s formula interprets as smaller crystallites. This is supported by the corresponding increase in micro-strain and dislocation density at this temperature, indicating the introduction of new structural defects or phase boundaries. Examining the lattice parameters and unit cell volume, the lattice parameters (a and c) and unit cell volume (V) show subtle but meaningful variations. The sample calcined at 700 °C (BiFe_0.9_Cu_0.1_O-700) possesses the largest unit cell volume (377.69 Å³). The c/a ratio, which indicates the degree of tetragonal distortion in the perovskite structure, also peaks for the 700 °C sample. This suggests that this calcination temperature promotes a specific lattice distortion, which could be linked to more effective Cu²⁺ incorporation (whose ionic radius is different from Fe³⁺) or a specific configuration of oxygen vacancies that strains the lattice. Most critically for photocatalytic application, the Specific Surface Area (SSA) shows a clear and important trend: it decreases as crystallites grow from 500 °C to 700 °C. This is direct evidence that the particles are becoming larger and denser, reducing the surface area available for dye adsorption and reaction. The subsequent increase in SSA for the 800 °C sample further supports the agglomeration hypothesis, as agglomerated particles often have a higher surface area due to interstitial spaces compared to dense, sintered crystals. These structural parameters create a compelling narrative for the observed photocatalytic activity. The sample calcined at 500 °C exhibits the optimal combination of properties: a small crystallite size, the highest specific surface area (38.1 m²/g), and a sufficiently high level of micro-strain. While higher temperatures improve crystallinity, they also lead to grain growth and a significant loss of surface area, which is detrimental to surface-dependent reactions like photocatalysis. Therefore, the superior performance of the 500 °C sample can be directly attributed to this synergistic balance of nanoscale dimensions, high surface area, and a favorable defect structure.

The lattice parameters (a and c) and unit cell volume (V) for the samples calcined at different temperatures are listed in Table 1. It was observed that the lattice parameter a and the unit cell volume (V) exhibited a similar trend with calcination temperature, showing only minor variations. This behavior is consistent with reports from other researchers^31,32^. The slight deviations in the lattice parameters can be attributed to changes in particle size and quantum size effects^33^, as well as the presence of point defects, dislocation defects, or oxygen vacancies. A clear trend is observed where the SSA decreases with increasing calcination temperature up to 700 °C, then increases at 800 °C. This trend inversely correlates with the crystallite size (D) shown in Table 1.

The structural parameters detailed in Tables 1 and 2, such as the contrasting micro-strain values between the two phases (Table 2), suggest the presence of a strained interface between them.

**Fig. 2:**
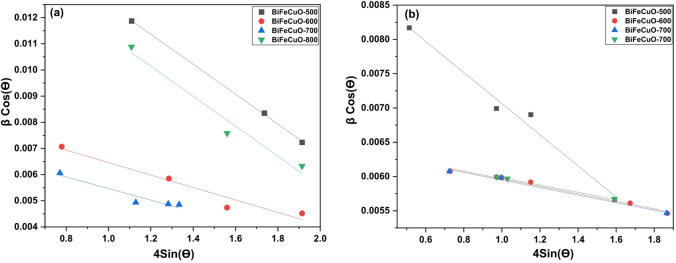
Williamson-Hall (W-H) plots for BiFe₀.₉Cu₀.₁O₃ nanoparticles calcined at different temperatures for (**a**) rhombohedral and (**b**) orthorhombic structure.

Furthermore, the Williamson-Hall (W-H) analysis was employed to deconvolute the contributions of crystallite size and microstrain to the peak broadening, using the equation:

8 $$\:\beta\:\mathrm{c}\mathrm{o}\mathrm{s}\theta\:=\frac{k\lambda\:}{D}+4\epsilon\:\mathrm{s}\mathrm{i}\mathrm{n}\theta\:$$

where β is the full width at half maximum (FWHM) in radians, θ is the diffraction angle, k is the shape factor (0.9), λ is the X-ray wavelength, D is the crystallite size, and ε is the microstrain. A plot of βcosθ versus 4sinθ was used for this analysis as shown in Fig. 2.^34,35^.

**Table 2:** A comparison of crystallite size (D) and micro-strain (ε) values for BiFe₀.₉Cu₀.₁O₃ samples calcined at different temperatures, as determined by the scherrer and Williamson-Hall (W-H) methods.

Sample	Crystal structure	Scherrer method		Williamson-Hall method	
		D (nm)	ε	D (nm)	ε
BiFeCuO-500	Rhombohedral	18.84	0.612	7.94	0.00572
	Orthorhombic	22.68	0.740	15.52	0.00227
BiFeCuO-600	Rhombohedral	23.87	0.524	16.38	0.00238
	Orthorhombic	24.35	0.578	22.20	0.000548
BiFeCuO-700	Rhombohedral	27.53	0.413	18.85	0.00222
	Orthorhombic	25.65	0.562	22.27	0.00055
BiFeCuO-800	Rhombohedral	20.61	0.571	8.51	0.00573
	Orthorhombic	25.01	0.632	22.24	0.000529

Table 2 provides a comparative analysis of the crystallite size (D) and micro-strain (ε) for the BiFe₀.₉Cu₀.₁O₃ samples, calculated using both the Scherrer and Williamson-Hall (W-H) methods. A key observation is the systematic discrepancy between the two methods: for a given phase and temperature, the Scherrer method consistently yields smaller crystallite sizes and significantly higher micro-strain values compared to the W-H analysis. This discrepancy is expected and highlights the fundamental difference between the two techniques. The Scherrer equation attributes peak broadening solely to crystallite size, thereby interpreting all broadening effects as a size contribution, which results in an overestimation of strain. In contrast, the W-H method deconvolutes the contributions of size and strain to peak broadening. The consistently lower strain values from the W-H plot suggest that the Scherrer-derived strain is overestimated. The data also reveals material-specific trends. The micro-strain in the orthorhombic (Bi₂Fe₄O₉) phase is notably lower than in the rhombohedral (BFO) phase across all samples when measured by the W-H method, indicating a more relaxed crystal structure for the secondary phase. Furthermore, the anomalous behavior of the sample calcined at 800 °C is clearly visible, where the W-H method shows a sharp increase in strain for the rhombohedral phase, corroborating the XRD findings that suggest significant lattice distortion or defect formation at this temperature Fig 2.

### Elemental composition and morphology investigation

**Fig. 3:**
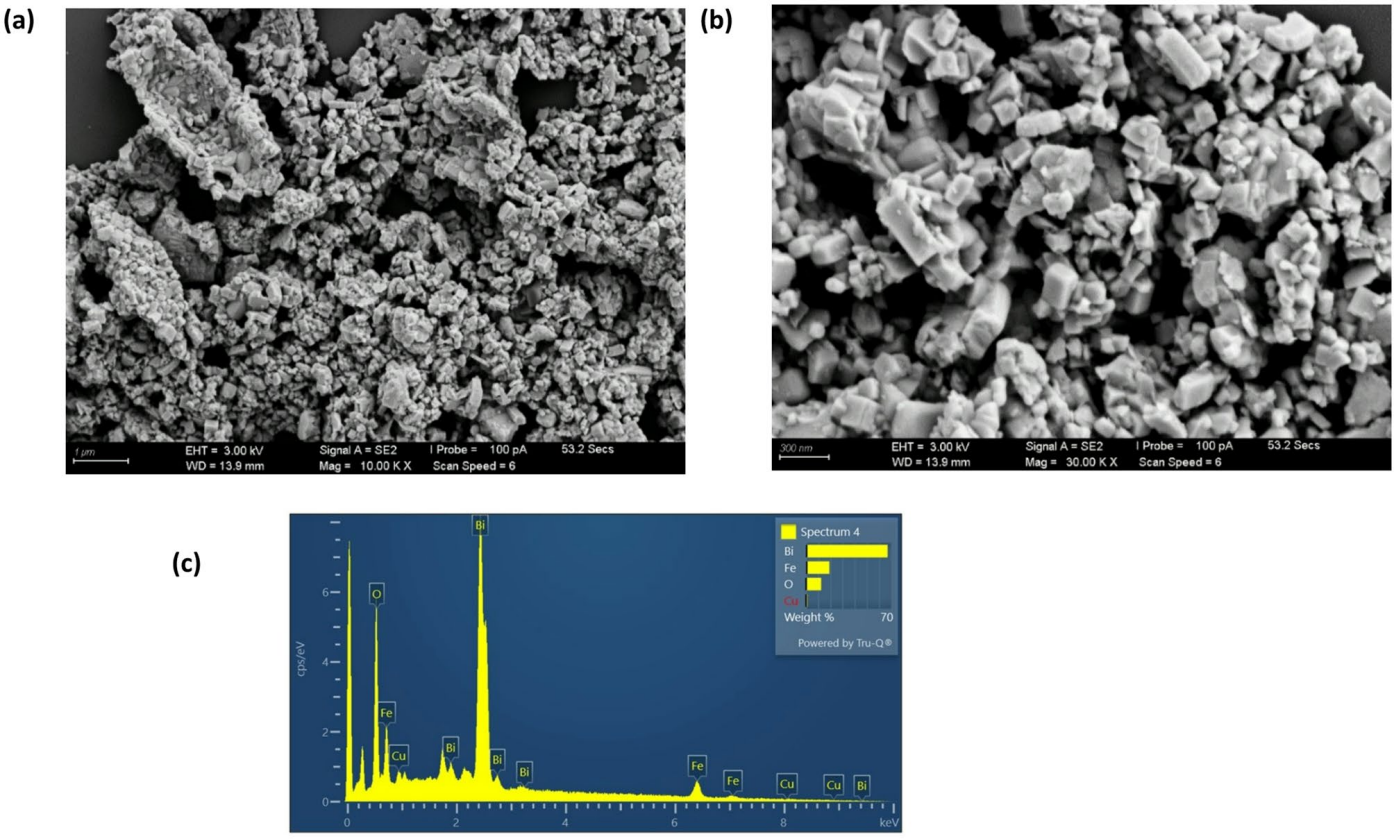
(**a**,** b**) FESEM images of BiFe₀.₉Cu₀.₁O₃ nanoparticles calcined at 600 °C at (**a**) low and (**b**) high magnification; (**c**) corresponding EDX spectrum confirming elemental composition.

The surface morphology and chemical composition of the 0.1 at% Cu-doped BiFeO₃ nanoparticles calcined at 600 °C were examined using field emission scanning electron microscopy (FESEM) and energy-dispersive X-ray (EDX) spectroscopy, respectively. Figure 3a, b presents FESEM images at two different magnifications. At lower magnification (Fig. 3a), the sample exhibits a mixed morphology consisting of nanospheres, nanorods, and nanoplates that have aggregated into larger, irregular structures. The higher magnification image (Fig. 3b) reveals that these primary nanostructures are composed of finer nanoparticles that have coalesced, indicating a tendency for agglomeration at the calcination temperature used.

EDX analysis was performed to verify the chemical composition and successful incorporation of copper. The resulting spectrum, shown in Fig. 3c, confirms the presence of bismuth (Bi), iron (Fe), copper (Cu), and oxygen (O) as the only elemental components, with no detectable impurities. The measured atomic percentages were found to be 21.89% for Bi, 23.56% for Fe, 1.28% for Cu, and 53.27% for O. The Cu content is very close to the nominal doping level of 0.1 at%, confirming the effectiveness of the synthesis process in incorporating the dopant into the material.

**Fig. 4:**
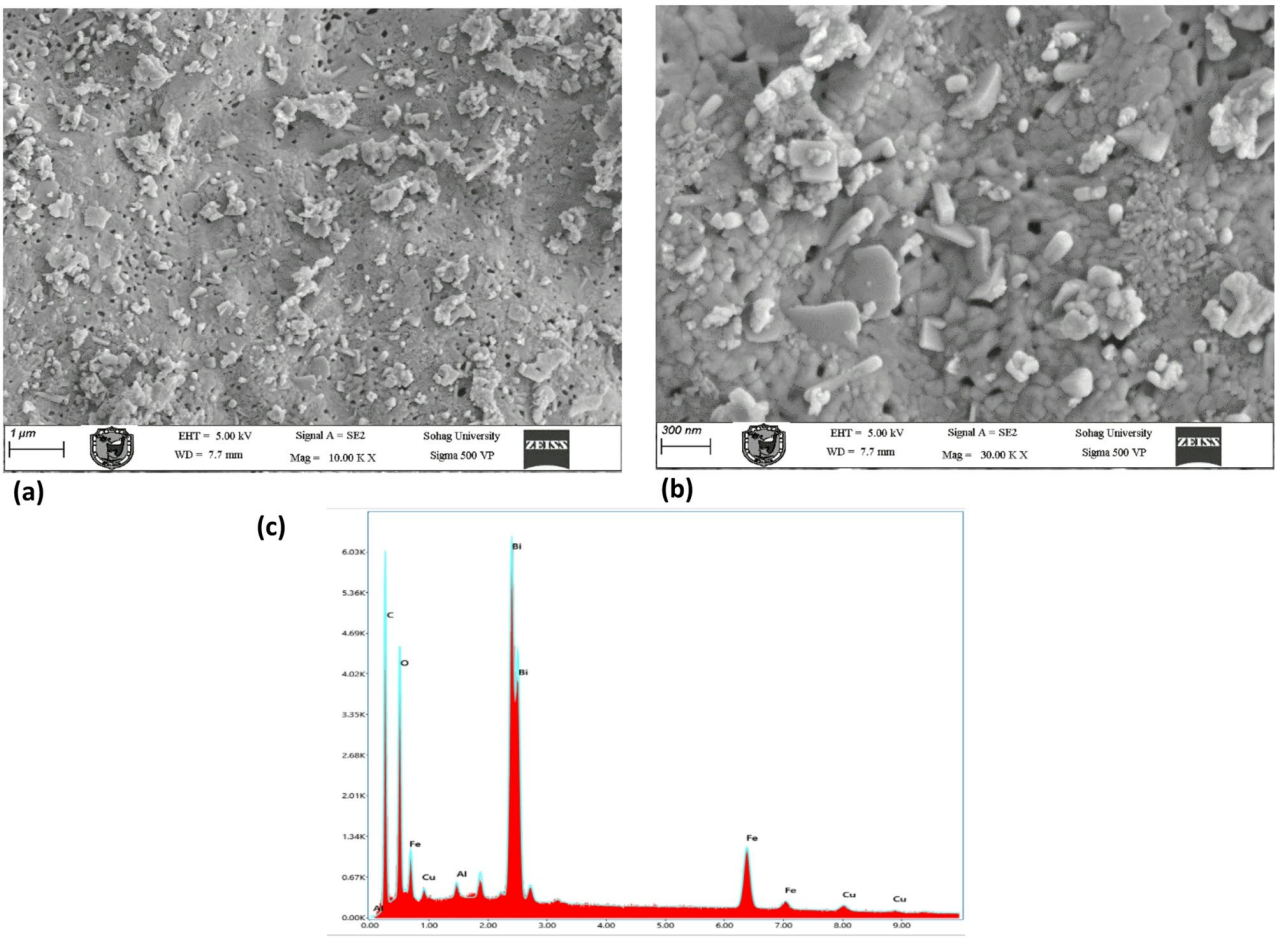
FE-SEM images of BiFe₀.₉Cu₀.₁O₃ nanoparticles calcined at 500 °C: (**a**) Low-magnification and (**b**) high-magnification FE-SEM images. (**c**) Corresponding EDX spectrum confirming the presence of Bi, Fe, Cu, and O, alongside trace artifacts of C and Al.

The microstructure of the BiFe₀.₉Cu₀.₁O₃ nanoparticles, calcined at 500 °C, was elucidated using field-emission scanning electron microscopy (FE-SEM). Figure 4a presents a low-magnification overview, revealing a primary surface that is dense and compact, yet punctuated by a network of mesopores. Notably, this surface is overlaid with a secondary layer of accumulated particulate agglomerates. A higher-magnification image (Fig. 4b) provides a more intricate view of the compact matrix, within which distinct embedded nanostructures are clearly resolved. These include nanorods, nanowalls, and nanosheets, indicating a complex growth morphology. Significantly, an analogous nanostructural evolution was observed in samples calcined at the higher temperature of 600 °C, suggesting this multi-scale architecture is a persistent feature of the synthesized material across this thermal range. Complementary energy-dispersive X-ray (EDX) spectroscopy (Fig. 4c) confirms the presence of the expected constituent elements: Bi, Fe, Cu, and O. The measured atomic percentages are 4.2%, 4.7%, 0.9%, and 23.8%, respectively. The spectrum also detected aluminum (0.3%) and a significant carbon signal (66.1%). The carbon is likely adventitious, originating from the environment or the sample preparation process, while the trace aluminum may be an artifact from the sample stub. The associated percentage errors for each element are provided, with the highest uncertainty (45%) noted for the low-concentration copper signal.

### FTIR analysis

**Fig. 5:**
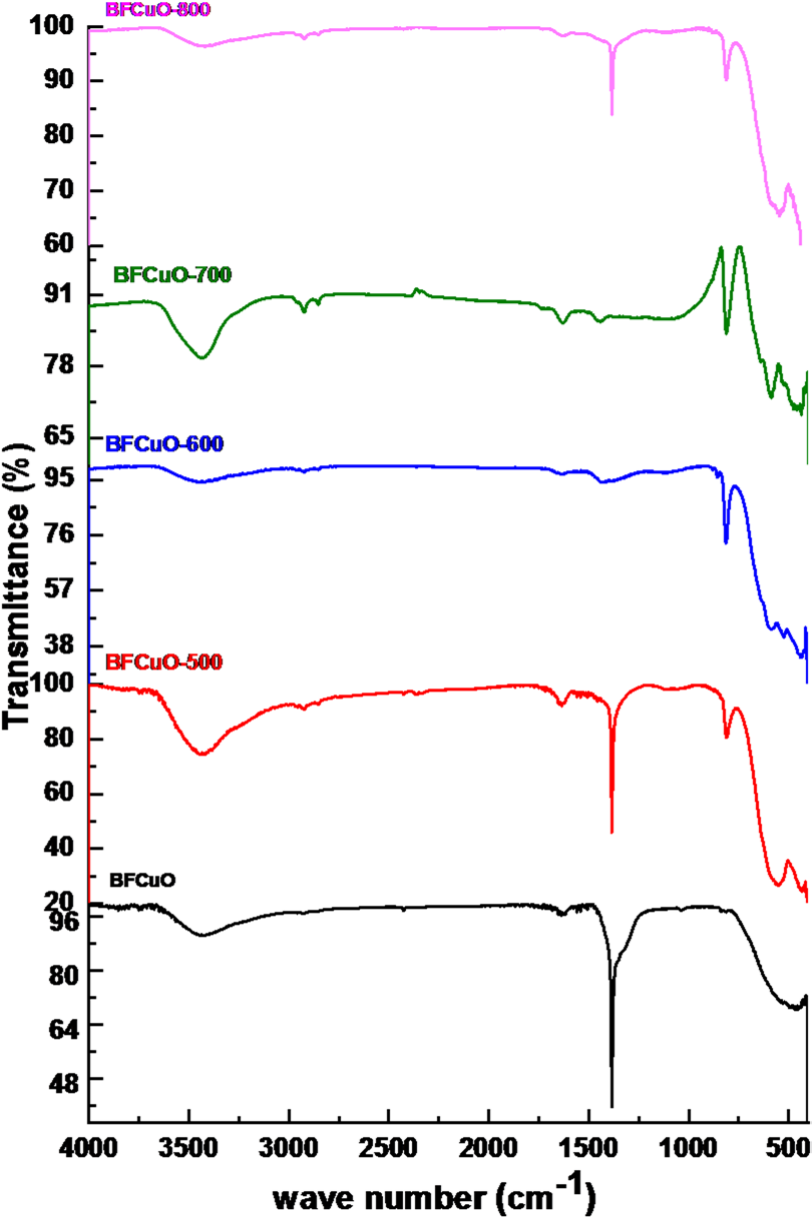
FTIR spectra of as-prepared and calcined BiFe₀.₉Cu₀.₁O₃ nanoparticles recorded from 400 to 4000 cm⁻¹.

The Fourier transform infrared (FTIR) technique was performed to study the vibrational mode properties of BiFe₀.₉Cu₀.₁O₃ nanoparticles. Figure 5 exhibits the FTIR spectra in the wavenumber range of 400–4000 cm⁻¹ for the as-prepared and calcined BiFe₀.₉Cu₀.₁O₃ nanoparticles. Absorption bands in the range of 400–1000 cm⁻¹ are related to metal-oxygen bonds, such as Fe-O, Bi-O, and Cu-O bonds^36,37^. The band positions and number of absorption peaks are dependent on the crystalline structure, chemical composition, and particle morphology^37^. Absorption bands around 465 and 550 cm⁻¹ are attributed to stretching vibrations of Fe-O and bending vibrations of O-Fe-O in FeO₆ groups in the perovskite structure^38^. Further, small peaks in the range of 400–600 cm⁻¹ give a clear signature of C-C and M-C-O bonding, which indicates the presence of a very small amount of carbonaceous materials remaining from the fuel combustion process^39^. On the other hand, the band around 815 cm⁻¹ corresponds to the bending vibration modes of Cu-O, while the strong peak around 1400 cm⁻¹ is also attributed to the vibration of the Bi-O bond^40^. Finally, the broad absorption band in the range of 3000–3600 cm⁻¹ is related to the O-H stretching vibrations, indicating the presence of adsorbed water on the sample^33^. It is also observed that there is a slight shift of the peaks to lower wavenumber with an increase in the annealing temperature. This shift can be related to a change in the lattice parameters of the BiFe₀.₉Cu₀.₁O₃ nanoparticles^33^.

### Surface area

**Fig. 6:**
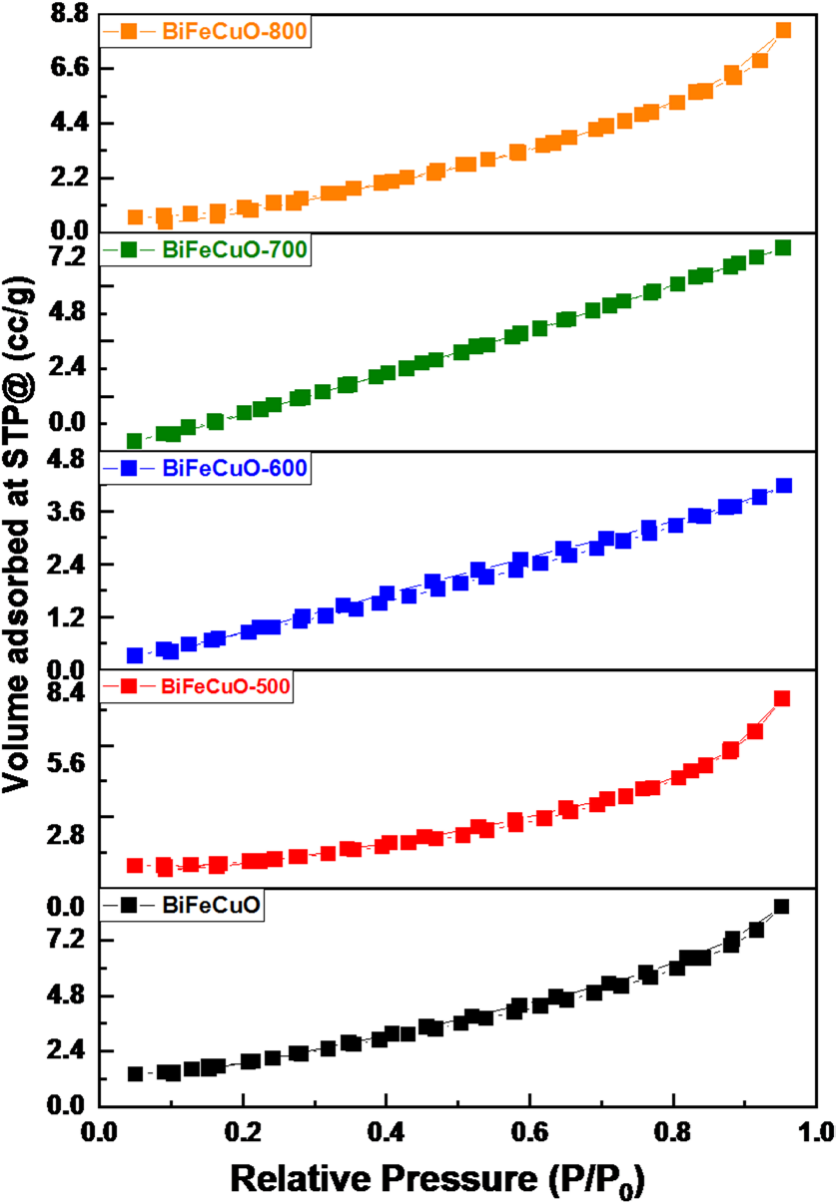
N₂ adsorption-desorption isotherms for BiFe₀.₉Cu₀.₁O₃ nanoparticles calcined at different temperatures.

The textural properties of the as-synthesized and calcined BiFe₀.₉Cu₀.₁O₃ samples, as determined by N₂ physisorption analysis, are crucial for understanding their photocatalytic potential. The BET-specific surface area (S_BET_), total pore volume, and average pore radius are summarized in Table 3. A clear trend is observed in the specific surface area with calcination temperature. The as-synthesized sample has a moderate surface area of 7.68 m²/g. Upon calcination at 500 °C, the surface area increases significantly to a maximum of 13.34 m²/g. This initial increase can be attributed to the combustion of residual organic fuels and the formation of a porous network during the crystallization of the previously amorphous material. However, as the calcination temperature is further increased to 600 °C and 700 °C, the surface area drops sharply to 4.85 m²/g and 4.01 m²/g, respectively. This is a classic symptom of sintering and crystallite growth, where smaller particles coalesce into larger, denser aggregates with reduced overall surface area. Interestingly, the sample calcined at 800 °C shows a slight recovery in surface area to 5.24 m²/g. This could be due to the initiation of particle fracturing or the growth of a secondary phase (like Bi₂Fe₄O₉) with a different morphology, creating new interparticle pores. The nitrogen adsorption-desorption isotherms, shown in Fig. 6, provide further insight. All samples exhibit a Type IV isotherm, which is characteristic of mesoporous materials (pores between 2 and 50 nm). The presence of a hysteresis loop in these isotherms confirms the capillary condensation of nitrogen within the mesopores. The shape of the hysteresis loop is consistent with an H3-type, often associated with slit-shaped pores formed by the aggregation of plate-like particles, which aligns with the mixed morphology observed in FESEM images.

**Table 3:** BET surface area, pore volume, and pore size data for the as-synthesized BiFe₀.₉Cu₀.₁O₃ perovskite and samples calcined at different temperatures.

Sample	Specific surface area S_BET_ (m²/g)	Total pore volume (cc/g)×10^− 2^	Average pore radius (Å)
BiFe_0.9_Cu_0.1_O_3_	7.68	1.339	19.723
BiFe_0.9_Cu_0.1_O_3_ -500	13.34	1.37	17.092
BiFe_0.9_Cu_0.1_O_3_ -600	4.85	0.6446	17.381
BiFe_0.9_Cu_0.1_O_3_ -700	4.01	1.149	17.208
BiFe_0.9_Cu_0.1_O_3_ -800	5.24	1.261	19.843

The pore structure data in Table 3 support this interpretation. The total pore volume and average pore radius do not follow a simple linear trend with temperature. Still, the sample calcined at 500 °C possesses a high pore volume (1.37 × 10⁻² cc/g) and a relatively small pore radius (17.1 Å). This combination of high surface area and a well-developed mesoporous structure is highly beneficial for photocatalysis, as it provides numerous active sites and facilitates the diffusion of reactant molecules. The subsequent decrease in surface area at higher temperatures directly correlates with a loss of these textural properties, which would be expected to diminish photocatalytic performance. Therefore, the 500 °C sample is texturally the most favorable, possessing the highest surface area and a favorable porous structure, which directly explains its superior photocatalytic activity as reported.

### XPS analysis

**Fig. 7:**
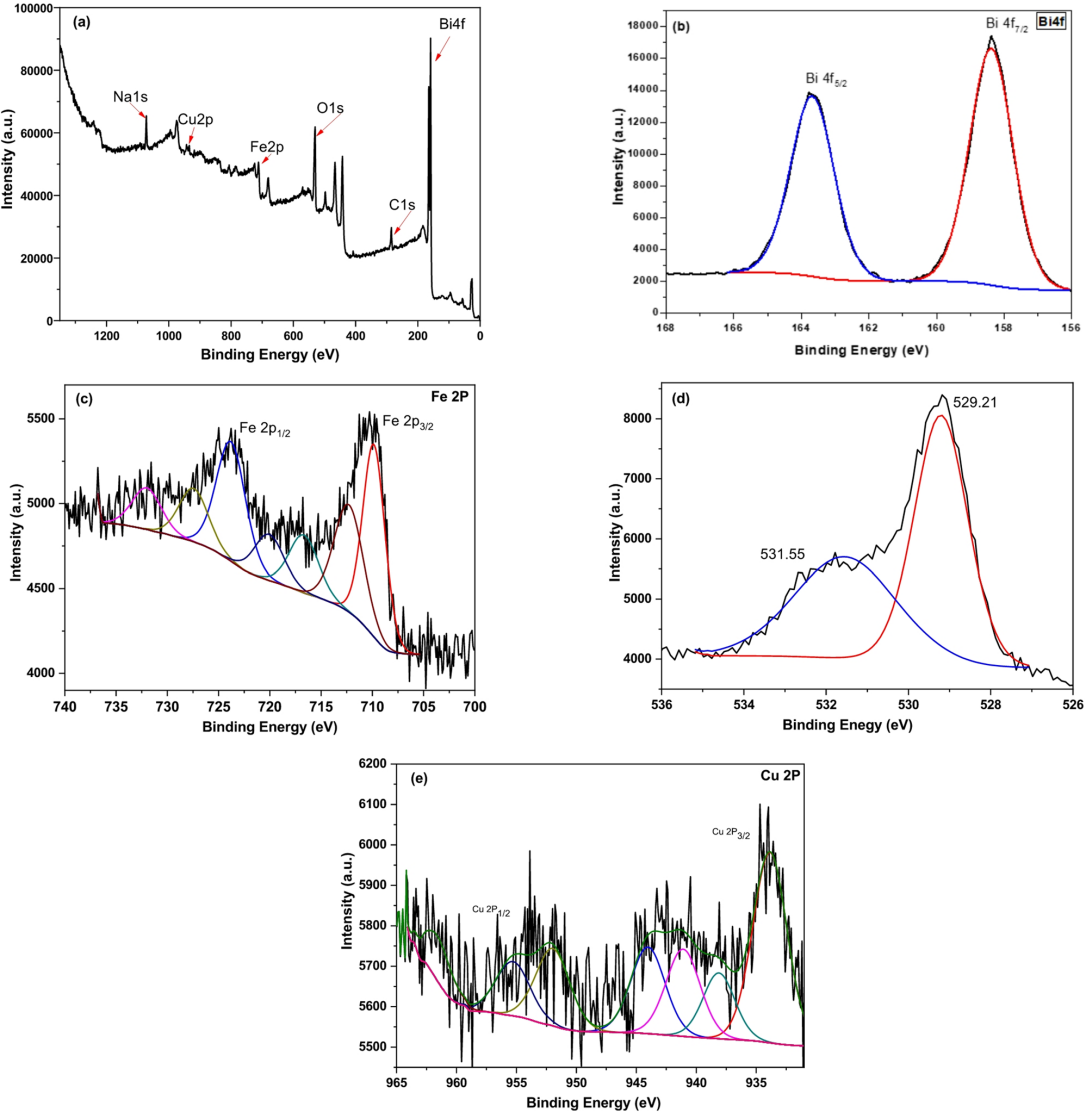
XPS spectra of BiFe_0.9_Cu_0.1_O_3_-600 ͦ C (**a**) survey spectra, (**b**) Bi 4f, (**c**) Fe 2p, (**d**) O 1s, and (**e**) Cu 2p orbitals of 1.0 mol% Cu-BiFeO_3_ nanoparticles.

X-ray photoelectron spectroscopy (XPS) was performed to determine the chemical composition and oxidation states of the elements in the 0.1% Cu-doped BiFeO₃ nanomaterials. The survey spectrum for the sample calcined at 600 °C, shown in Fig. 7a, confirms the presence of Bi, Fe, O, and Cu, with the C 1s peak at 284.8 eV used as a reference for charge correction. The high-resolution spectra for each element are presented in Fig. 7b-e. The Bi 4f spectrum (Fig. 7b) displays two distinct peaks at binding energies of 158.4 eV and 163.7 eV, corresponding to the Bi 4f_7/2_ and Bi 4f_5/2_ states, respectively. This doublet confirms the presence of bismuth in its trivalent state (Bi³⁺) within the perovskite lattice^40,41^.

As shown in Fig. 7c, the Fe 2p spectrum is characterized by two broad peaks at 709.8 eV (Fe 2p_3/2_) and 723.6 eV (Fe 2p_1/2_), indicative of Fe–O bonds. The presence of a satellite peak located approximately 8 eV above the main Fe 2p_3/2_ peak, at 717.2 eV, is a definitive signature of the Fe³⁺ oxidation state^42^. The O 1s spectrum (Fig. 7d) was deconvoluted into two components: a lower binding energy peak at 529.2 eV, attributed to lattice oxygen (metal-oxygen bonds), and a higher binding energy peak at 531.6 eV, which is associated with surface-adsorbed oxygen species or oxygen vacancies^42^. The Cu 2p spectrum (Fig. 7e) shows the characteristic spin-orbit doublet with peaks at 933.8 eV (Cu 2p_3/2_) and 955.1 eV (Cu 2p_1/2_). The positions and the absence of strong shake-up satellite features confirm that copper is present in the + 2 oxidation state (Cu²⁺) and is successfully incorporated into the BiFeO₃ structure^43,44^.

### Magnetic properties

**Fig. 8:**
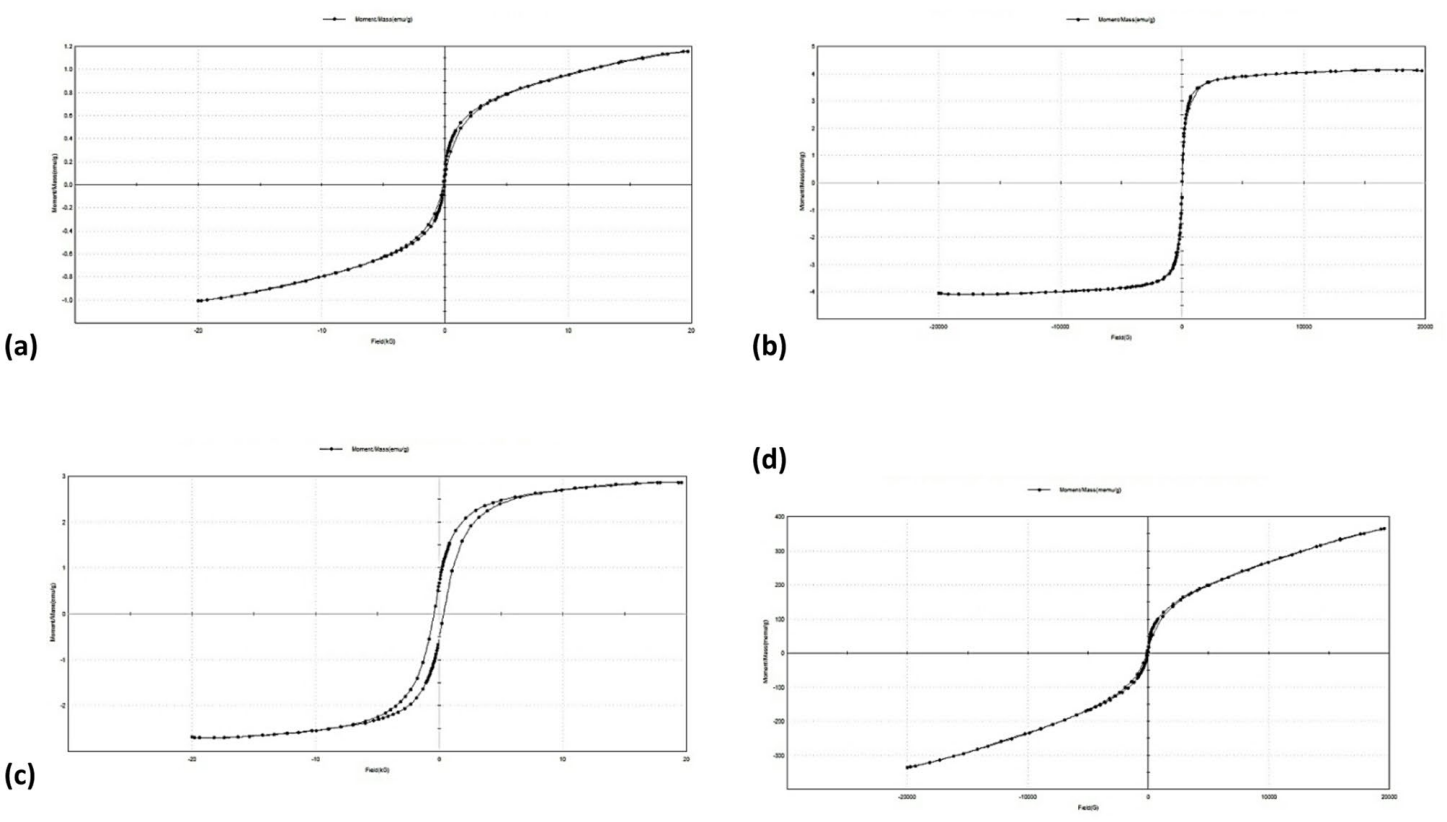
Hysteresis loop (M versus H) of BiFe_0.9_Cu_0.1_O_3_ calcined at (**a**) 500, (**b**) 600, (**c**) 700, and (**d**) 800 ͦ C.

The magnetic behavior of BiFe₀.₉Cu₀.₁O₃ nanoparticles calcined at different temperatures was investigated using a vibrating sample magnetometer (VSM), with the resulting hysteresis loops presented in Fig. 8a-d. The observation of clear hysteresis cycles at room temperature confirms that all samples exhibit ferromagnetic (FM) behavior, which represents a significant departure from the antiferromagnetic (AFM) character of bulk, pure BiFeO₃^45^. This transition from AFM to FM in nanostructured BFO is a well-known phenomenon, primarily attributed to two key factors: the breakdown of the long-range spin cycloid and the introduction of structural defects^46–48^. The magnetic parameters extracted from these loops, saturation magnetization (M_s_), remanent magnetization (M_r_), and coercive field (H_c_), are summarized in Table 4 and reveal a complex, non-monotonic dependence on calcination temperature. The most pronounced trend is the dramatic evolution of M_s_. The value increases sharply from 1.082 emu/g at 500 °C to a maximum of 4.108 emu/g at 600 °C, then declines to 2.78 emu/g at 700 °C and falls precipitously to 0.35 emu/g at 800 °C. This behavior can be explained by the interplay between several competing mechanisms. The initial increase in M_s_ up to 600 °C is driven by the enhanced crystallinity and the effective suppression of the spatially modulated spin spiral structure. Bulk BFO-Cu possesses an incommensurate spin cycloid with a period of ~ 62 nm. When the particle size, as influenced by calcination, becomes comparable to or smaller than this period, the spiral is disrupted, leading to a net uncompensated magnetic moment and the emergence of ferromagnetism. Concurrently, the synthesis process and Cu²⁺ doping are known to generate oxygen vacancies (Vₒ). These vacancies play a dual role: they break the superexchange pathways (Fe³⁺–O–Fe³⁺) that mediate antiferromagnetism, and they can induce localized changes in the valence state of iron (e.g., the formation of Fe⁴⁺ to maintain charge balance), both of which create uncompensated spins and enhance the overall magnetization^49–52^. The peak Mₛ at 600 °C suggests this temperature provides the optimal balance between improved crystallinity and a high concentration of these magnetically active defects.

However, as the calcination temperature increases further to 700 °C and 800 °C, the saturation magnetization declines. This decrease is attributed to several co-occurring phenomena. Firstly, significant particle growth and agglomeration occur, as confirmed by XRD and FESEM, which reduces the surface-to-volume ratio. Since surface atoms often have a higher degree of spin canting and uncompensation, their reduced proportion diminishes their contribution to the net magnetization. Secondly, at higher temperatures, there is a tendency for the annihilation of oxygen vacancies as the lattice approaches a more thermodynamically stable state. This “healing” of defects reduces the number of broken superexchange pathways and uncompensated spins. Furthermore, the growth of the secondary orthorhombic Bi₂Fe₄O₉ phase, which is paramagnetic or weakly magnetic at room temperature, acts as a magnetic diluent within the studied samples, lowering the measured M_s_ of the entire system^53^. The coercivity (H_c_) also displays a remarkable variation, decreasing from 53.95 G at 500 °C to a mere 3.91 G at 700 °C, before increasing again to 45.87 G at 800 °C. This trend is fundamentally linked to the magnetic domain state and microstructural evolution. The high H_c_ at 500 °C is characteristic of single-domain magnetic nanoparticles, which require a substantial energy barrier to reverse their magnetization uniformly. As the crystallite size increases with calcination temperature (from 500 °C to 700 °C), the particles likely surpass the single-domain critical size and transition into a multi-domain state. In multi-domain particles, magnetization reversal proceeds through the relatively low-energy process of domain wall motion, resulting in a much lower coercivity, as seen at 600 °C and 700 °C. The subsequent recovery of H_c_ at 800 °C is intriguing and may be caused by the emergence of a multi-domain structure where domain wall motion is pinned by defects, grain boundaries, or the interfaces between the primary BFO and the secondary Bi₂Fe₄O₉ phase, making magnetization reversal more difficult and thus increasing the coercive field^54^.

The magnetic properties of the Cu-doped BFO nanoparticles are not governed by a single factor but are a delicate consequence of the calcination-induced trade-off between crystallite size, oxygen vacancy concentration, phase purity, and magnetic domain evolution. The sample calcined at 600 °C achieves the optimal condition, maximizing the saturation magnetization through effective spiral suppression and a favorable defect profile.

**Table 4:** Magnetic saturation (M_s_), remanent magnetization (M_r_), and coercive field (H_c_) as a function of calcination temperature.

Calcination temperature	M_s_	H_c_	M_*r*_
500 ͦ C	1.082	53.95	0.18
600 ͦ C	4.108	6.76	1.8
700 ͦ C	2.78	3.91	0.8
800 ͦ C	0.35	45.87	0.050

### Photoluminescence analysis

**Fig. 9:**
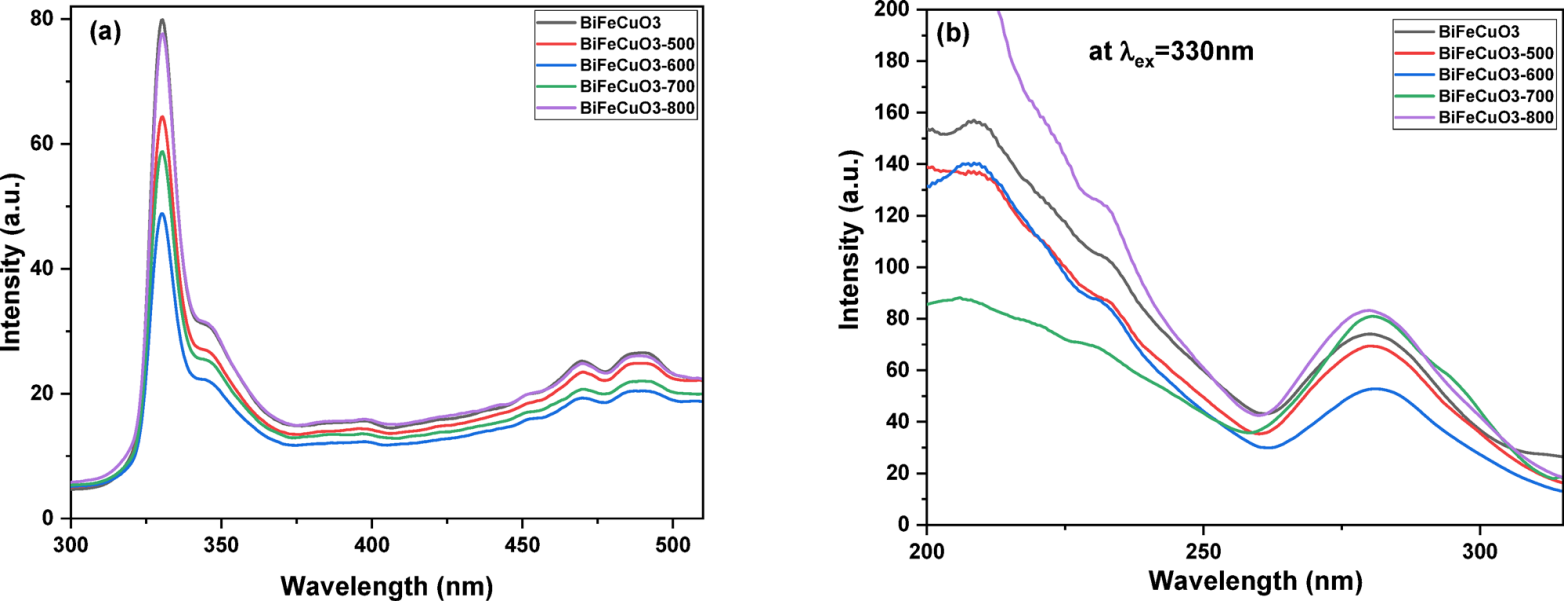
Photoluminescence (PL) spectra of BiFe₀.₉Cu₀.₁O₃ nanoparticles and calcined products (**a**) Emission spectrum monitored at an excitation wavelength (λ_ex_) of 330 nm; (**b**) Excitation spectrum monitored at the emission wavelength (λ_em_) of 330 nm.

Photoluminescence (PL) spectroscopy is a powerful technique for probing the electronic structure, imperfections, and recombination dynamics of photogenerated charge carriers in semiconductor materials. The intensity of the PL signal is directly related to the rate of radiative recombination of electron-hole pairs; therefore, a lower PL intensity typically indicates a higher separation efficiency of charge carriers, which is a desirable property for photocatalytic applications. Figure 9a, b shows the PL spectra acquired at room temperature for the as-synthesized and calcined BiFe₀.₉Cu₀.₁O₃ nanocrystallites. The spectra reveal that BiFe₀.₉Cu₀.₁O₃ nanocrystallites exhibited a strong blue emission band in the range of 200 to 310 nm, centered around 280 nm (approximately 4.43 eV), when excited at 330 nm. This high-energy emission is typically associated with near-band-edge transitions but can also be influenced by the presence of defect states, such as oxygen vacancies, which are known to create localized energy levels within the band gap. The variation in PL intensity among the calcined samples provides critical insight into the calcination-dependent density of these defect sites and their role as recombination centers.

### Diffuse reflectance spectroscopy

**Fig. 10:**
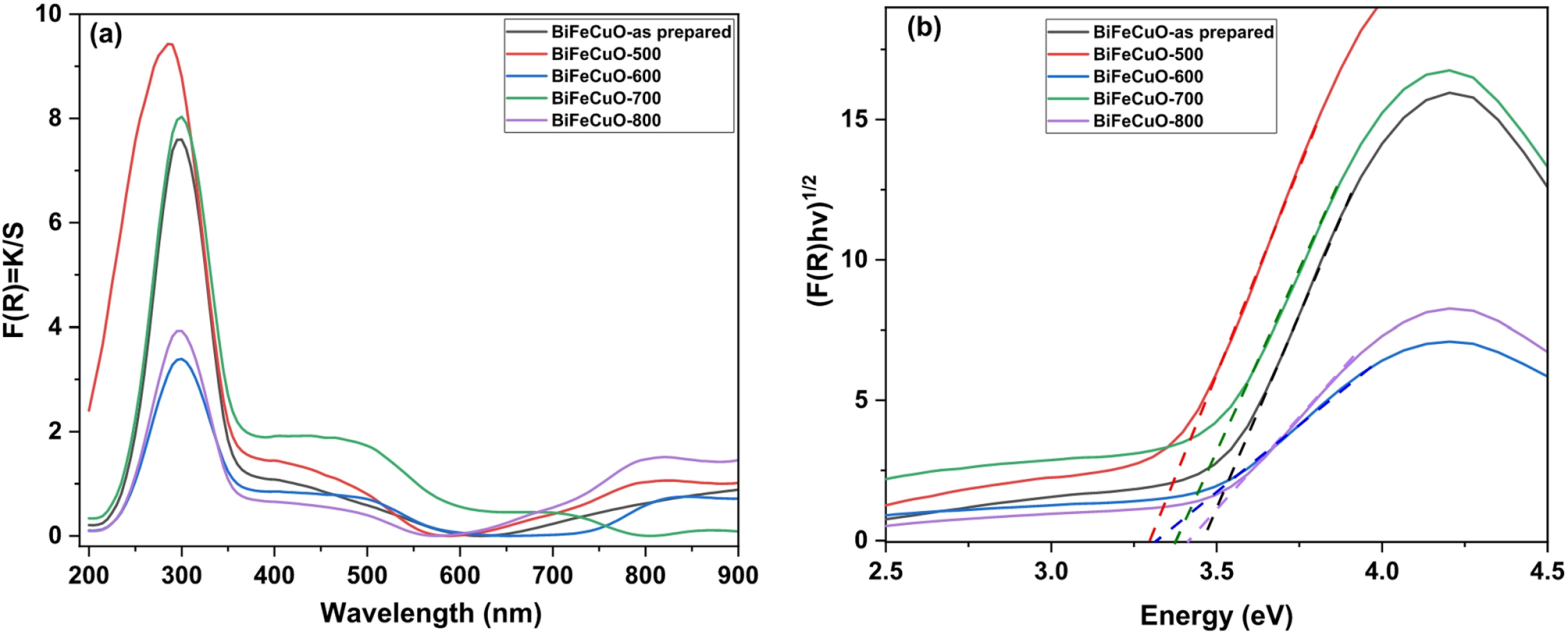
(**a**) UV-Vis reflectance spectrum of as-synthesized and calcined BiFe_0.9_Cu_0.1_O_3_ nanoparticles, (**b**) (F(R)hυ)^1/2^ VS hυ of as-synthesized and calcined samples.

Diffuse reflectance spectroscopy (DRS) was used to characterize the optical properties of the BiFe₀.₉Cu₀.₁O₃ nanocrystallites. The reflectance data were analyzed using the Kubelka-Munk theory to determine the absorption characteristics, as described by the function F(R) = (1 - R)²/2R, where R is the reflectance, F(R) is proportional to the absorption coefficient, and the scattering factor is assumed to be constant^55^. Figure 10a shows the plot of the Kubelka-Munk function F(R) versus the wavelength for the BiFe₀.₉Cu₀.₁O₃ nanocrystallites. It was observed that the as-synthesized BiFe₀.₉Cu₀.₁O₃ nanocrystallites had an absorption peak at 297 nm, which was blue-shifted to 285 nm for the sample calcined at 500 °C, and red-shifted to 298 nm for the sample calcined at 700 °C. The optical band gap energy (E_g_) was determined using the Tauc relation: [F(R)hν]^n^ = A(hν - E_g_), where A is a constant, h is Planck’s constant, ν is the frequency, and the exponent n denotes the nature of the electronic transition. For BiFeO₃-based materials, which are typically direct band gap semiconductors, *n* = 1/2 was used for direct allowed transitions^3^. Accordingly, the value of [F(R) × hν]^1/2^ was plotted against the photon energy (hν) as shown in Fig. 9b. The optical band gap (E_g_) was determined by extrapolating the linear portion of the curve to the x-axis. The deduced direct optical band gap E_g_ values are listed in Table 5.

It is observed that the E_g_ value for the as-synthesized sample is 3.45 eV, which decreases to a minimum of 3.30 eV for the sample calcined at 500 °C. With a further increase in calcination temperature to 700 °C, the band gap increases to 3.37 eV. This initial decrease in E_g_ can be attributed to improved crystallinity and a reduction in the concentration of defects that create trap states within the band gap, leading to a more defined absorption edge^56^. The subsequent increase in E_g_ at higher temperatures (700–800 °C) is consistent with the decrease in crystallite size observed at 800 °C in XRD analysis, suggesting a strengthening of the quantum confinement effect^57,58^. These results agree with the findings of other researchers^59^. Furthermore, the band edge positions for the conduction band (E_CB_) and valence band (E_VB_) were calculated to understand the photocatalytic potential. The empirical equations based on electronegativity were used^60^:

9 $$E_{{VB}} = {\text{ }}\chi - E_{e} + 0.5E_{g}$$

10 $$E_{{CB}} = {\text{ }}E_{{VB}} - E_{g}$$

Here, χ is the absolute electronegativity of BiFe₀.₉Cu₀.₁O₃, calculated to be 5.88 eV, and E_e_ is the energy of free electrons on the hydrogen scale (~ 4.5 eV). The calculated E_VB_ and E_CB_ values for all samples are listed in Table 5. The positive valence-band positions confirm the strong oxidizing potential of the photogenerated holes, while the negative conduction-band positions indicate a strong reducing power for the electrons, which is favorable for photocatalytic redox reactions.

**Table 5:** Optical band gap (E_g_), Valence band (E_VB_), and conduction band (E_CB_) energies for as-prepared and calcined BiFe₀.₉Cu₀.₁O₃ samples.

Sample	E_g_	E_VB_	E_CB_
BiFeCuO	3.45	3.13	-0.32
BiFeCuO-500	3.3	3.055	-0.245
BiFeCuO-600	3.31	3.06	-0.25
BiFeCuO-700	3.37	3.09	-0.28
BiFeCuO-800	3.41	3.11	-0.3

### Photocatalytic degradation

**Fig. 11:**
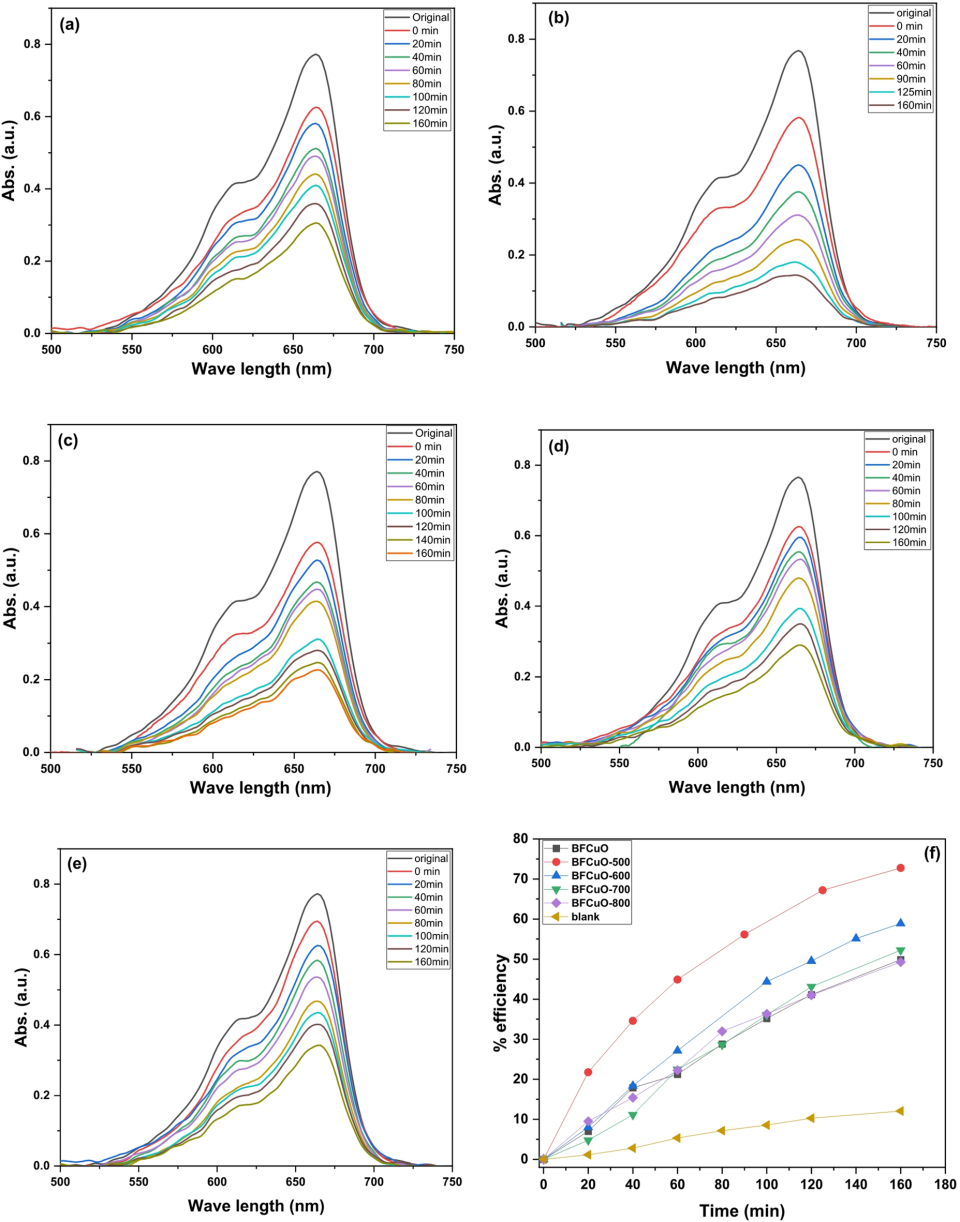
(**a**-**e**) Temporal evolution of MB absorption spectra under visible-light irradiation for BiFe₀.₉Cu₀.₁O₃ nanoparticles at (**b**) 500 °C, (**c**) 600 °C, (**d**) 700 °C, and (**e**) 800 °C; (**f**) Corresponding photocatalytic degradation efficiency versus irradiation time.

A control experiment conducted in the absence of the photocatalyst showed no appreciable degradation, confirming that the reaction is photocatalytically driven. The temporal evolution of the MB absorption spectrum under visible-light irradiation is shown in Fig. 11a-e for all samples. The characteristic absorption peak of MB at λ_max_ ≈ 664 nm decreases in intensity with increasing irradiation time, with the most rapid and pronounced decrease observed for the BiFe₀.₉Cu₀.₁O₃-500 sample. Figure 11f. illustrates the photocatalytic degradation efficiency of methylene blue (MB) dye versus irradiation time for the as-prepared and calcined BiFe₀.₉Cu₀.₁O₃ nanoparticles. The degradation efficiency (%) was calculated using the equation:

11 $$Degradation{\text{ }}efficiency{\text{ }}\left( \% \right){\text{ }} = {\text{ }}\left[ {\left( {C_{0} {\text{ }} - {\text{ }}C} \right){\text{ }}/{\text{ }}C_{0} } \right]{\text{ }} \times {\text{ }}100~$$

where C₀ is the initial concentration of the MB dye, and C is the concentration at a given irradiation time. The results demonstrate a strong dependence of photocatalytic performance on the calcination temperature. The efficiency increases with annealing temperature, reaching a maximum for the sample calcined at 500 °C, after which a decreasing trend is observed at higher temperatures. The degradation percentages after 160 min were found to be 49.9%, 72.75%, 58.9%, 52.2%, and 49.3% for the as-synthesized sample and those calcined at 500, 600, 700, and 800 °C, respectively.

The photocatalytic activity is governed by two primary competing factors: crystallite size and specific surface area. Generally, a smaller crystallite size and a larger specific surface area provide more active sites for the adsorption and reaction of dye molecules, leading to higher photocatalytic activity. This is consistent with the XRD and BET data, which show that the sample calcined at 500 °C possesses the smallest crystallite size and the highest specific surface area, directly correlating with its superior performance (72.75% degradation). Furthermore, the introduction of oxygen vacancies (Vₒ, Vₒ⁺, Vₒ⁺⁺) at lower calcination temperatures (up to 500 °C) plays a crucial role. These defects act as electron traps, effectively suppressing the recombination of photogenerated electron-hole pairs, thereby increasing the availability of charge carriers for the redox reactions that degrade the dye^61,62^. The decrease in photocatalytic activity at calcination temperatures above 500 °C is attributed to several factors. Primarily, particle agglomeration and crystallite growth, as confirmed by XRD and FESEM, lead to a significant reduction in specific surface area^63^. Additionally, the increased presence of the secondary orthorhombic Bi₂Fe₄O₉ phase, as identified in the XRD analysis, can act as recombination centers for photogenerated charge carriers, further reducing efficiency^56^. The as-synthesized sample, despite its high surface area, exhibits lower activity due to its amorphous nature, which contains a high density of defects and imperfections that promote rapid charge carrier recombination^56^.

**Fig. 12:**
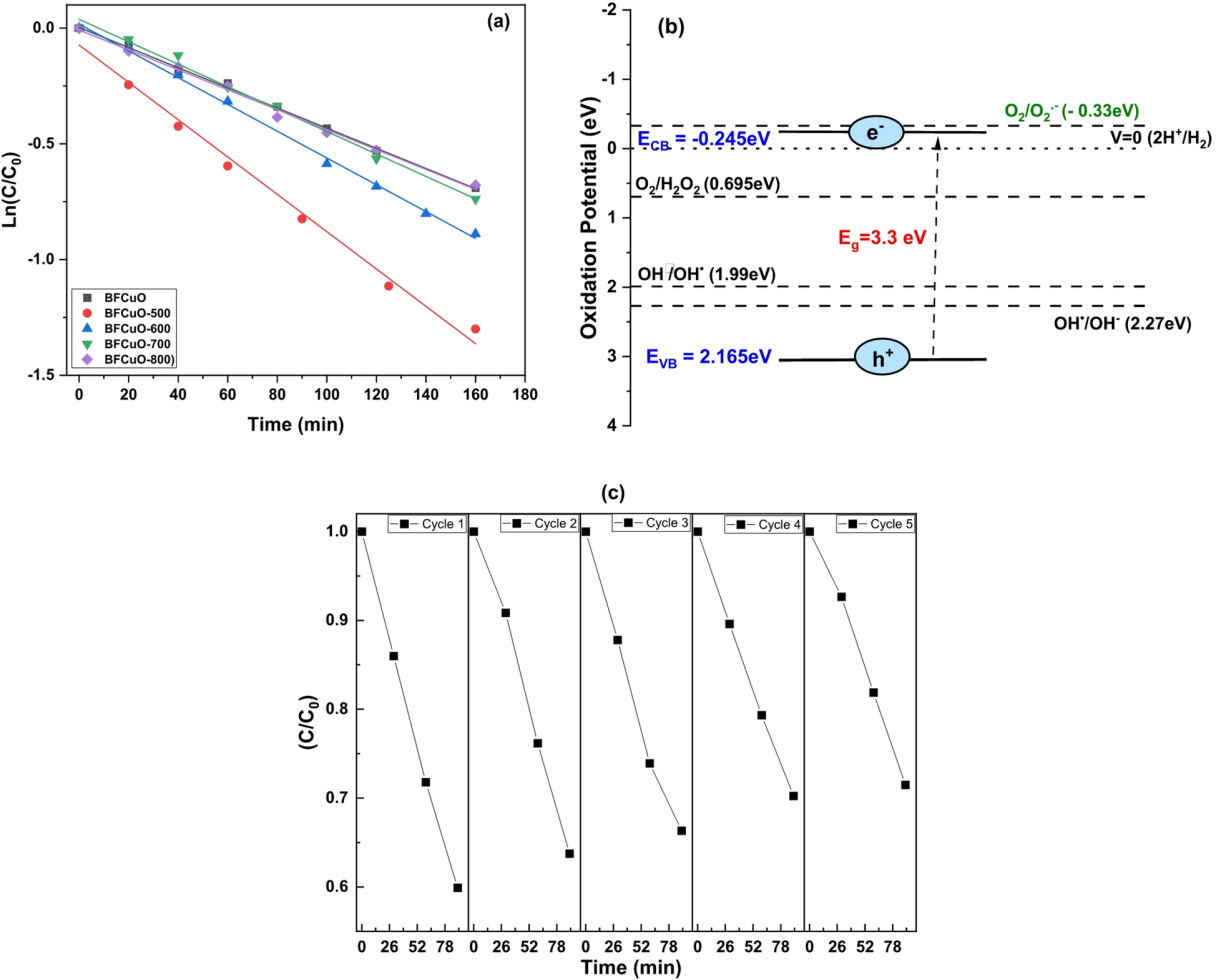
(**a**) Pseudo-first-order kinetics of MB photodegradation. (**b**) Proposed photocatalytic mechanism for BiFe₀.₉Cu₀.₁O₃-500. (**c**) Catalyst reusability over five cycles.

The reaction kinetics were analyzed using a pseudo-first-order model based on the Langmuir-Hinshelwood mechanism^64^:

12 $$\ln \left( {C_{0} /C} \right){\text{ }} = {\text{ }}kt~$$

,where k is the apparent rate constant. The linear plots of ln(C₀/C) versus irradiation time are shown in Fig. 12a. The calculated rate constants (k) for the as-synthesized sample and those calcined at 500, 600, 700, and 800 °C are 0.0046, 0.0092, 0.0058, 0.0051, and 0.0049 min⁻¹, respectively. The sample calcined at 500 °C possesses the highest rate constant, confirming it as the most active photocatalyst. A proposed mechanism for the photocatalytic degradation is illustrated in Fig. 12b. Upon visible light irradiation with energy greater than the band gap, electron-hole pairs are generated in the BiFe₀.₉Cu₀.₁O₃ catalyst (Eq. 13). The photogenerated electrons (e⁻) in the conduction band can react with adsorbed oxygen molecules to form superoxide radical anions (•O₂⁻) (Eq. 14). Simultaneously, the photogenerated holes (h⁺) in the valence band can oxidize water (H₂O) or surface hydroxyl groups (OH⁻) to produce highly reactive hydroxyl radicals (^•^OH) (Eqs. 15 & 16). These reactive oxygen species (•O₂⁻ and •OH) are the primary agents responsible for the oxidative degradation of the MB dye molecules into harmless end products like CO₂ and H₂O (Eqs. 17 & 18). However, considering the band edge positions from Table 5 (E_CB_ = -0.245 eV for the 500 °C sample), the reduction of O₂ to •O₂⁻ (E⁰ = -0.33 eV vs. NHE) is thermodynamically less favorable. In this case, the oxygen vacancies can play a critical role by facilitating the alternative pathway of electron capture, preventing recombination, and allowing the holes to directly or indirectly (via ^•^OH formation) oxidize the dye^65^. The stability and reusability of the BiFe₀.₉Cu₀.₁O₃-500 photocatalyst, crucial for practical applications, were evaluated over five consecutive cycles, as shown in Fig. 12c. The catalyst exhibited excellent stability, with the degradation efficiency decreasing by only about 12% after the fifth run. This minimal loss in activity confirms the robust nature and high reusability potential of the synthesized photocatalyst for the treatment of organic pollutants.

The results demonstrate that the calcination temperature of 500 ͦ C is the optimum temperature for achieving the maximum photocatalytic degradation of MB dye in aqueous solution under visible light irradiation.

13 $$BiFe_{{0.9}} Cu_{{0.1}} O_{3} - 500{\text{ }} + {\text{ }}h\upsilon {\text{ }} \to {\text{ }}BiFe_{{0.9}} Cu_{{0.1}} O_{3} - 500{\text{ }}\left( {e^{ - } + {\text{ }}h^{ + } } \right)$$

14 $$e^{ - } + O_{2} ^{ - } \to {\text{ }}O_{2} ^{{ \cdot ^{ - } }} ~$$

15 $$h^{ + } + H_{2} O{\text{ }} \to {\text{ }}OH^{ \bullet } + H^{ + } ~$$

16 $$OH^{ - } + {\text{ }}h^{ + } \to {\text{ }}OH^{ \bullet } ~$$

17 $$OH^{ \bullet } + MB{\text{ }} \to {\text{ }}CO_{2} + {\text{ }}H_{2} O{\text{ }}\left( {\deg radation{\text{ }}products} \right)$$

18 $$O_{2} ^{{ \bullet ^{ - } }} + MB{\text{ }} \to {\text{ }}CO_{2} + {\text{ }}H_{2} O{\text{ }}\left( {\deg radation{\text{ }}products} \right)$$

### Effect of scavengers

**Fig. 13:**
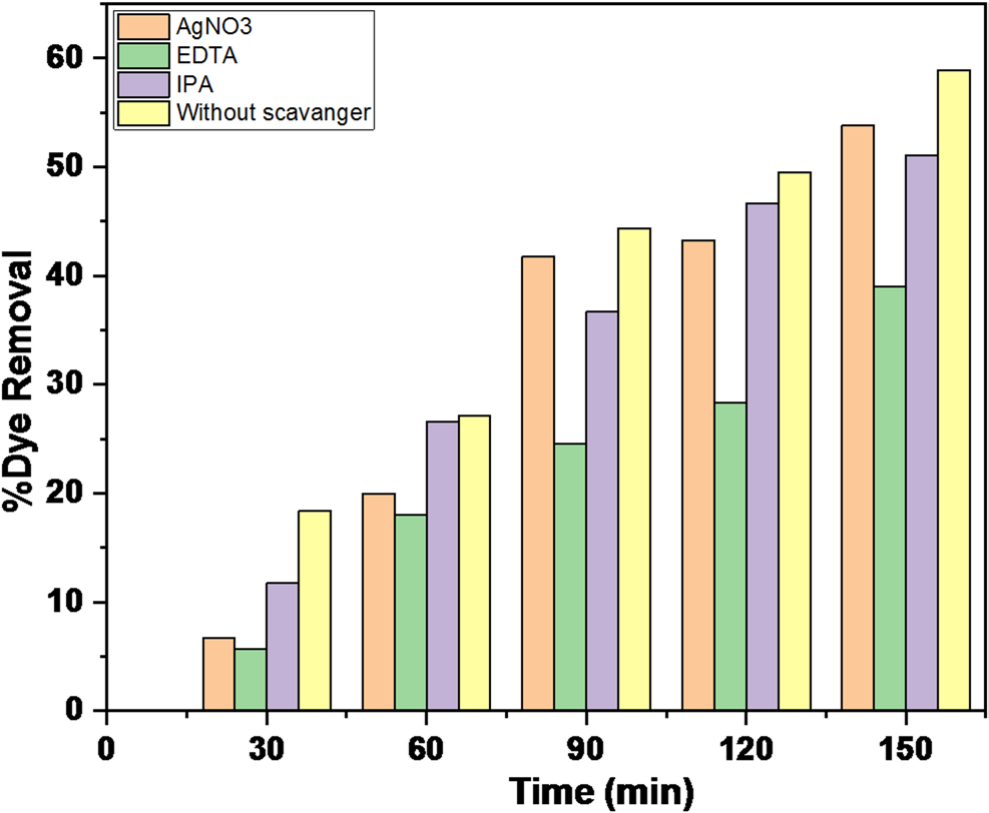
Effect of different radical scavengers on the photocatalytic degradation efficiency of MB, demonstrating the dominant role of photogenerated holes (h⁺).

To examine the roles of different active species involved in the photocatalytic degradation of MB dye, control and trapping experiments were performed using BiFe_0.9_Cu_0.1_O_3_ calcined at 600 °C. In the trapping experiment, EDTA (hole scavenger), AgNO_3_ (electron scavenger), and isopropanol (HO^•^ scavenger) were introduced to the solution before starting the photocatalytic reaction, and the results are shown in Fig. 13. For the test with 1 mmol of AgNO_3_, no significant inhibition was observed, which supported the conclusion from the conduction band potential calculations. This suggested a less negative conduction band value, meaning that electrons in the conduction band could not contribute to the formation of superoxide radicals. In contrast, the addition of 1 mmol of EDTA led to a noticeable inhibition of the degradation process, while 10 mmol of isopropanol caused only a slight inhibition. This difference is due to EDTA blocking the holes (h+), thereby preventing the formation of HO• radicals, whereas isopropanol only blocked the hydroxyl radicals (HO•). Consequently, no significant degradation occurred. These results indicate that holes (h+) are the primary species responsible for MB degradation, as they drive the production of HO• radicals (Eq. 15). On the other hand, electrons (e-) seem to have minimal impact, as capturing them did not result in a notable change in degradation activity.

The scavenger tests provide crucial insight into the primary reactive species responsible for the photocatalytic degradation mechanism. The results indicate that the degradation of the dye proceeds through multiple pathways, with photogenerated holes (h⁺) playing the most dominant role. This conclusion is drawn from the significant decrease in dye removal efficiency when Ethylenediaminetetraacetic acid (EDTA), a known hole scavenger, was added to the reaction. The efficiency dropped dramatically from ~ 58.9% to 39.0%, representing the largest inhibition among all scavengers tested. This strong suppression confirms that reactions initiated by holes are the most critical for the degradation process, either through direct oxidation of the dye molecules or via the formation of secondary oxidative species.

The other scavengers showed a less pronounced, but still notable, effect. The addition of AgNO₃ (a scavenger for electrons, e⁻) reduced the efficiency to ~ 53.8%, indicating that free electrons in the conduction band also contribute to the process, likely by reducing molecular oxygen to form superoxide radicals (•O₂⁻). The addition of Isopropanol (IPA, a scavenger for hydroxyl radicals, •OH) reduced efficiency to ~ 51.1%, suggesting that •OH radicals play a relatively minor role in this specific photocatalytic system. The modest impact of IPA implies that the standard pathway of hole-mediated formation of •OH radicals from water/OH⁻ is not the primary route. Instead, the direct oxidation by holes and the reduction of oxygen by electrons to form •O₂⁻ appear to be the more dominant mechanisms. This mechanistic understanding is consistent with the band structure analysis, which suggested that the conduction band potential might be suitable for O₂ reduction while the valence band potential is highly positive, favoring direct hole oxidation.

## Conclusion

BiFe₀.₉Cu₀.₁O₃ nanoparticles were successfully synthesized via a simple solution combustion method and subsequently calcined at different temperatures. A comprehensive investigation of the structural, vibrational, optical, magnetic, and photocatalytic properties revealed a strong dependence on the calcination temperature. XRD analysis confirmed that the as-synthesized powder was amorphous, while calcination produced a primary rhombohedral BiFeO₃ phase alongside a secondary orthorhombic Bi₂Fe₄O₉ phase. The average crystallite size generally increased with calcination temperature, reaching a maximum at 700 °C (27.53 nm) before a slight decrease at 800 °C due to potential agglomeration or secondary phase growth. Conversely, the specific surface area was highest for the sample calcined at 500 °C. The optical band gap exhibited a complex trend, decreasing to a minimum of 3.30 eV at 500 °C due to improved crystallinity and defect reduction, before increasing again at higher temperatures, likely influenced by quantum confinement effects and phase changes. Magnetic measurements revealed ferromagnetic behavior at room temperature, with the saturation magnetization peaking at 600 °C (4.108 emu/g) due to the effective suppression of the spin spiral structure and the presence of oxygen vacancies, before declining at higher temperatures as a result of particle growth and vacancy annihilation. The photocatalytic performance for methylene blue degradation under visible light was maximized for the sample calcined at 500 °C, achieving 72.75% degradation in 160 min. This optimal activity is attributed to a synergistic combination of a small crystallite size, the highest specific surface area (providing abundant active sites), a favorable band gap for visible light absorption, and a beneficial concentration of oxygen vacancies that suppresses electron-hole recombination. The decline in performance at higher calcination temperatures was directly correlated with increased particle agglomeration, reduced surface area, and the growing influence of the secondary Bi₂Fe₄O₉ phase acting as charge recombination centers. Therefore, the solution combustion synthesis followed by calcination at 500 °C is established as the optimal process for producing Cu-doped BFO nanoparticles with superior structural, optical, and textural properties for efficient photocatalytic environmental remediation.

## Data Availability

All data generated or analyzed during this study are included in this article, and any other data will be made available on request from the corresponding author.
